# Enhancing Early Detection of Diabetic Foot Ulcers Using Deep Neural Networks

**DOI:** 10.3390/diagnostics15161996

**Published:** 2025-08-09

**Authors:** A. Sharaf Eldin, Asmaa S. Ahmoud, Hanaa M. Hamza, Hanin Ardah

**Affiliations:** 1 Department of Information and Decision Support Systems, Faculty of Information Technology and Computer Science, Sinai University, Arish 16020, Egypt; ahmed.sharaf@su.edu.eg; 2 Department of Information Systems, Faculty of Computers and AI, Helwan University, Cairo 11795, Egypt; 3 Department of Information Technology, Faculty of Information Technology and Computer Science, Sinai University, Arish 16020, Egypt; 4 Department of Information Technology, Faculty of Computers and Informatics, Zagazig University, Zagazig 44519, Egypt; hmkamal@fci.zu.edu.eg; 5 Department of Computer Sciences, College of Computer and Information Sciences, Princess Nourah bint Abdulrahman University, P.O. Box 84428, Riyadh 11671, Saudi Arabia; hyabdullrahman@pnu.edu.sa

**Keywords:** diabetic foot, diabetic foot ulcers (DFUs), deep neural network (DNN), deep learning (DL), thermal images, plantar thermograms

## Abstract

**Background/Objectives**: Diabetic foot ulcers (DFUs) remain a critical complication of diabetes, with high rates of amputation when not diagnosed early. Despite advancements in medical imaging, current DFU detection methods are often limited by their computational complexity, poor generalizability, and delayed diagnostic performance. This study presents a novel hybrid diagnostic framework that integrates traditional feature extraction methods with deep learning (DL) to improve the early real-time computer-aided detection (CAD) of DFUs. **Methods**: The proposed model leverages plantar thermograms to detect early thermal asymmetries associated with DFUs. It uniquely combines the oriented FAST and rotated BRIEF (ORB) algorithm with the Bag of Features (BOF) method to extract robust handcrafted features while also incorporating deep features from pretrained convolutional neural networks (ResNet50, AlexNet, and EfficientNet). These features were fused and input into a lightweight deep neural network (DNN) classifier designed for binary classification. **Results**: Our model demonstrated an accuracy of 98.51%, precision of 100%, sensitivity of 98.98%, and AUC of 1.00 in a publicly available plantar thermogram dataset (*n* = 1670 images). An ablation study confirmed the superiority of ORB + DL fusion over standalone approaches. Unlike previous DFU detection models that rely solely on either handcrafted or deep features, our study presents the first lightweight hybrid framework that integrates ORB-based descriptors with deep CNN representations (e.g., ResNet50 and EfficientNet). Compared with recent state-of-the-art models, such as DFU_VIRNet and DFU_QUTNet, our approach achieved a higher diagnostic performance (accuracy = 98.51%, AUC = 1.00) while maintaining real-time capability and a lower computational overhead, making it highly suitable for clinical deployment. **Conclusions**: This study proposes the first integration of ORB-based handcrafted features with deep neural representations for DFU detection from thermal images. The model delivers high accuracy, robustness to noise, and real-time capabilities, outperforming existing state-of-the-art approaches and demonstrating strong potential for clinical deployment.

## 1. Introduction

**Figure 1:**
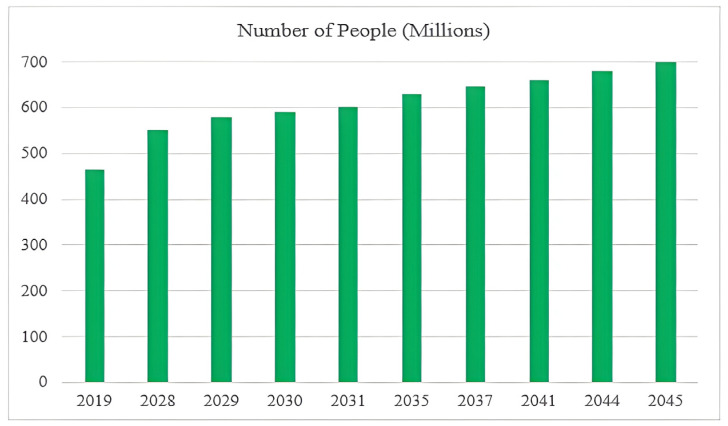
Projected global increase in diabetes and DFUs.

Diabetic foot disease (DF) is a major global health concern due to its high rates of morbidity, recurrence, and limb loss. Delayed diagnosis and management can lead to serious outcomes such as deep tissue infections, chronic ulceration, and partial or complete lower-limb amputation [1]. According to the International Diabetes Federation (IDF), approximately 463 million people were living with diabetes in 2019, a number projected to exceed 700 million by 2045. Among them, an estimated 9.1–26.1 million develop diabetic foot ulcers (DFUs) annually, with 15–25% expected to experience a DFU at some point in their lives [2], as shown in Figure 1. Over one million diabetic individuals with “high-risk feet” undergo amputation procedures each year due to complications from DFUs [3]. These ulcers are often difficult to detect at an early stage because symptoms can be subtle, and clinical diagnosis relies heavily on subjective judgment and physical examination.

This underscores the urgent need for accurate, objective, and early diagnostic tools. The proposed model in this study addresses these challenges by leveraging thermal imaging and artificial intelligence to enable non-invasive, real-time detection of DFUs based on subtle temperature anomalies—well before visible signs appear.

Traditional clinical scoring systems, such as the Wagner classification, PEDIS (Perfusion, Extent, Depth, Infection, and Sensation), and SINBAD (Site, Ischemia, Neuropathy, Bacterial infection, and Depth), are widely used to assess the severity of diabetic foot ulcers (DFUs). However, each framework has notable limitations. For example, the Wagner system is structurally simplistic, focusing solely on ulcer depth and ignoring critical factors such as ischemia and infection, which are essential for a comprehensive assessment [4,5]. The PEDIS classification offers a more detailed approach but still relies on subjective clinical judgment and manual scoring, which introduces inter-observer variability and can delay timely diagnosis [6]. Similarly, although the SINBAD score provides a simplified scoring tool suitable for clinical audits, it does not incorporate any imaging data and lacks predictive power for long-term outcomes [7].

To the best of our knowledge, no existing clinical classification system (Wagner, PEDIS, or SINBAD) currently supports automated, real-time, thermal-image-based DFU detection. These limitations emphasize the need for automated, objective, and real-time diagnostic methods. In this regard, thermal imaging and artificial intelligence (AI) provide promising directions. Unlike conventional scoring methods, AI-powered systems can automatically process plantar thermograms to detect early signs of DFUs based on subtle temperature asymmetries in the thermograms. The proposed hybrid model addresses these limitations by integrating both handcrafted ORB features and deep learning-based representations, offering a robust, accurate, and noninvasive tool for early DFU detection.

Early DFU identification is essential, but existing techniques (radiography, computed tomography (CT), and magnetic resonance imaging (MRI)) often require critical time, multiple hospital visits, and access to specialists. Thermal imaging is a non-invasive, radiation-free substitute that can identify minute temperature variations in the first stages of DFUs. This makes it a safe and accurate method for diagnosing medical problems [8]. However, there is a growing need for automatic, real-time diagnostic systems that can efficiently analyze these images.

Deep neural networks (DNNs), particularly convolutional models such as ResNet and Inception, can learn spatial and semantic features, making them ideal for DFU detection tasks [9]. Using DNNs, a computer-aided detection (CAD) system can detect DFUs early, even before symptoms appear on the skin. By leveraging convolutional layers to detect spatial features and utilizing pretrained architectures, such as ResNet-50, GoogLeNet, Inception, and Inception-ResNet which have been applied to medical imaging with high accuracy [10,11,12,13], these networks enable highly accurate classification of thermal images, streamlining early diagnosis processes. This could reduce the need for frequent visits to a specialist and possibly lower the risk of serious complications such as amputations.

The proposed model significantly enhances DFU detection by integrating ORB-based handmade characteristics with deep learning models. This establishes a precise, real-time method for locating DFUs. Our hybrid approach incorporates both low- and high-level characteristics, thereby enhancing its resilience to noise and image distortions compared with previous models. The main contributions of this study are as follows.

- An efficient and accurate hybrid diagnostic model combining ORB-based handcrafted feature extraction and a DNN model was proposed. It achieved a promising accuracy score compared with the other investigated models
- The proposed model is lightweight and consists of two hidden layers, each containing 32 neurons, making it suitable for real-time clinical applications.
- An analytical comparison of DF detection was conducted between the proposed model and the other investigated models (M. Adam et al. [14], M. Goyal et al. [15], Alzubaidi et al. [16], Khandakar et al. [17], Cruz-Vega et al. [18], Khandakar et al. [19], Reyes-Luévano et al. [20], and M. H. Alshayeji et al. [21]), as shown in Table 1. The results obtained are superior to those of other recently published methods.
- Extensive experiments were conducted using a publicly available plantar thermogram dataset [https://ieee-dataport.org/open-access/plantar-thermogram-database-study-diabetic-foot-complications, accessed on 1 December 2024], which includes thermal images of diabetic and non-diabetic individuals. The experimental results confirmed the model’s ability to generalize across the subjects and foot regions.

To the best of our knowledge, no prior study has integrated ORB-based handcrafted features with pretrained DNNs for DFU detection from thermal imagery, making our methodology novel.

The remainder of this paper is structured as follows: Section 2 discusses prior studies on the thermal imaging of diabetic feet. Section 3 describes the data acquisition and preprocessing methods used in this study. Section 4 details the proposed hybrid framework and the DNN model. Section 5 presents the results, discusses the limitations, and outlines future directions. Finally, Section 6 concludes the paper.

## 2. Related Work

This section presents an analysis of the previously developed DFU classification algorithms. M. Adam et al. [14] analyzed 33 thermographic images of the feet belonging to non-diabetics and type 2 diabetic patients. The authors used DWT [22] along with Higher-Order Spectra (HOS) [23] to decompose the images and extract relevant texture features. Their model, which included an SVM classifier based on a pool selection of only five crucial features, produced satisfactory results: sensitivity of 81.81%, accuracy of 89.39%, and specificity of 96.97%.

M. Goyal et al. [15] utilized machine learning to examine the disparities in diabetic foot ulcers compared to normal skin. The scientists used 292 images of diabetic foot ulcers and 105 images of healthy feet. Specialists annotated the data by manually delineating the ulcer area. The authors employed zero-centering and normalization techniques to standardize the data range. The authors used LBP [24] and HOG [25] to extract features that showed how texture and gradients varied between images. The authors proposed a new CNN, DFUNet, that distinguishes between healthy skin and DFUs using parallel layers to extract features at various levels. Using DFUNet, they achieved highly accurate classification with an F1-score of 93.9%. L. Alzubaidi et al. [16] proposed a DL model for automatically classifying healthy skin and DFUs using the model DFU_QUTNet. The authors used 754 color images from the diabetic center of Nasiriyah Hospital. A specialist labeled the images to help train the model. All images were 224 × 224 pixels, focusing on the ulcer area and surrounding tissue. The results for their method with DFU_QUTNet showed an F1-score of 93.24%; however, using an SVM classifier, they achieved a higher accuracy of up to 94.5%.

Cruz-Vega et al. [17] investigated the application of DL for classifying thermographic images of diabetic feet. The authors trained a convolutional neural network (CNN) architecture on thermal images collected from diabetic and non-diabetic patients, focusing on automated feature learning without manual intervention. Their results demonstrated that CNNs could effectively distinguish DFU patterns based on heat distribution, achieving competitive classification metrics. This study provides early evidence for the viability of thermal image-based DL models in real-time DFU screening systems.

Khandakar et al. [18] introduced a novel image-based machine learning approach for DFU detection by proposing the Thermal Change Index (TCI), which quantifies asymmetrical temperature variations between the feet. Using thermogram images, their method extracted features that reflect pathological heat distributions and fed them into various machine learning classifiers. This study demonstrated that incorporating physiological asymmetry via TCI significantly improved the diagnostic performance of the model. This study highlights how specific thermal descriptors rooted in clinical interpretation can enhance the explainability and robustness of DFU detection algorithms.

In an earlier study, Khandakar et al. [19] developed a traditional machine learning pipeline for early DFU detection using handcrafted features from foot thermograms. The authors extracted statistical and texture-based descriptors, such as mean temperature differences and GLCM features [26], and used these as inputs to classical classifiers, including SVMs and decision trees. The model was evaluated using a well-labeled thermogram dataset, achieving promising accuracy and sensitivity. This study underscores the potential of interpretable ML techniques in thermally based diabetic foot analysis, particularly in resource-constrained settings.

Simultaneously, Reyes-Luévano et al. [20] created a new method using estimation maps to identify high-risk areas of DFUs and suggested a deep learning model called DFU VIRNet to automatically determine the difference between normal skin and skin with DFUs. The model was trained using both visible and thermographic images. They focused on regions of interest (ROIs) resized to 150 × 150 pixels, capturing the key details of both healthy and ulcer-affected skin. A specialist classified these regions as normal or abnormal. DFU VIRNet outperformed existing methods, achieving an AUC of 99.301% and an accuracy of 97.75%. This study is particularly notable as it represents one of the most recent advances (2023) in combining visible and infrared modalities using deep learning to enhance DFU detection performance.

Recently, I. Khosa et al. [27] employed thermograms at both the image and patch levels for DFU detection. The authors employed various machine learning-based classification methods. For DFU recognition, they created their own features, such as HOG, Gabor [28], and the gray-level co-occurrence matrix (GLCM) [29]. In addition, the study evaluated the performance of two CNN models, ResNet50 and DenseNet121, in the recognition of DFUs. The authors introduced a custom lightweight CNN model proposed for automatic DFU recognition designed to work on multilevel thermographic data (image, patch, and combined levels). The proposed CNN-based model demonstrated superior performance compared to the employed models and their state-of-the-art counterparts, as evidenced by the higher AUC and accuracy values. Using image-level thermogram data instead of patch-level or combined image-patch thermograms also made both ML and DL methods more accurate in recognizing objects. As a 2023 contribution, this study reflects the emerging trend of using multilevel thermal representations and lightweight CNN architectures for more accessible and accurate DFU classification.

M. H. Alshayeji et al. [21] developed a conventional ML model for real-time CAD diagnosis of plantar hyperthermia using the plantar thermogram database. Preprocessing methods, such as min–max normalization [30] and CLAHE [31], were applied to standardize and enhance the image quality, making subtle details more visible. Feature extraction involves both traditional and DL methods. Techniques such as SIFT [32] and SURF [33] identify key points in images that remain consistent with changes in scale or rotation. These are organized using the Bag of Features (BOF) method [34], which clusters features into a dictionary of patterns, allowing images to be represented as simplified histograms. The study also employed DL models, such as ResNet-50, through transfer learning and adapted pretrained models to extract complex features from thermal images. Combining these features with support vector machine (SVM) classifiers enables the accurate classification of images into DFU or normal categories. To ensure robustness, this study used 10-fold cross-validation, which helps avoid overfitting and offers a dependable assessment of model performance. This recent study (2023) highlighted the effectiveness of hybrid handcrafted and deep features in thermal image analysis for early DFU recognition, which aligns closely with our research focus.

Table 1 summarizes the relevant studies from 2018 to 2023, including their methodologies, feature extraction strategies, classifiers, performance, and limitations. Notably, recent studies published post-2020 have demonstrated the growing application of deep learning and hybrid models in DFU detection. Compared with these previous approaches, our proposed method further advances the field by explicitly combining ORB-based efficiency, BOF robustness, and deep learning precision in a lightweight, real-time architecture.

## 3. Materials and Methods

In this section, we describe the datasets and methods used to build the proposed DNN model for diagnosing DFUs using the plantar thermal images.

### 3.1. DFU Dataset Collection and Preprocessing

This study employed a plantar thermogram database [35] that was specifically curated to develop and validate the proposed model. The plantar thermogram dataset explicitly used is available online in IEEE Data Port under DOI: [https://ieee-dataport.org/open-access/plantar-thermogram-database-study-diabetic-foot-complications, accessed on 1 December 2024]. The labels (“Normal” or “DFU”) were labeled by medical staff; thus, high reliability was guaranteed by the clear inter-observer agreements and quality control.

**Figure 2:**
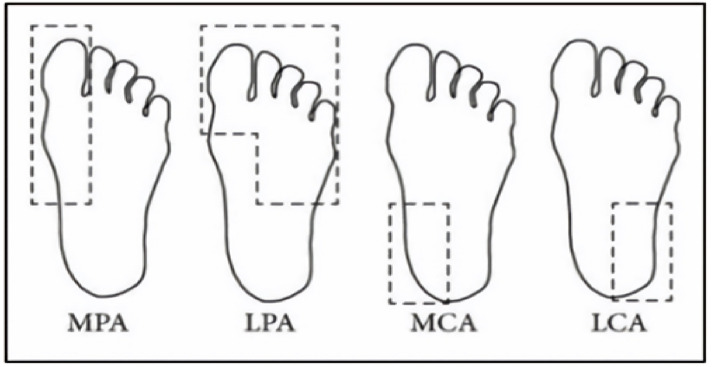
Plantar angiosomes [36].

The dataset consisted of thermal images captured from various regions of the plantar surface. A total of 122 subjects with diabetes and 45 without diabetes were included in this study. The corresponding comma-separated values (*. csv) file denotes the temperature of each pixel. Each folder contained a subfolder, with each subfolder housing four images representing the four plantar angiosomes of each foot. The angiosomes comprised the LPA, LCA, MPA, and MCA (Figure 2). The database consisted of 20 files per subject (10. png images and 10 .csv files), amounting to 1670 RGB thermal images and temperature values. The dataset explicitly collected ethical approval and informed consent from participants, which was provided in the dataset’s source documentation (IEEE Data-port). To ensure robust model performance, the data were divided into training (80%) and testing (20%) sets, maintaining a balanced representation of both DFU and normal cases. Class imbalance was directly addressed using stratified splitting (80% training, 20% testing) to obtain a balanced presentation.

We then applied min–max normalization and CLAHE as preprocessing steps to enhance the contrast of the thermal images. CLAHE is a powerful image enhancement technique that splits an image into small regions or tiles and applies histogram equalization to each tile, effectively enhancing the local contrast without over-amplifying noise in homogeneous areas. This method enhances the visibility of features in low-contrast areas, capturing even minor variations in texture and intensity, such as temperature differences in DFU diagnosis. In contrast to global histogram equalization, CLAHE prevents noise from becoming louder by clipping the histogram at a set value. This prevents areas with the same contrast from becoming too dark. This was achieved by enhancing the local contrast in each area. Table 2 displays the preprocessing outcomes for a set of sample thermal images (normal and DFUs).

### 3.2. Preliminary DFU Features Extraction

This study employed two distinct feature extraction techniques to enhance the classification of thermal images.

#### 3.2.1. Extracting Handcrafted Features

Handcrafted features are manually developed and extracted from raw data based on domain knowledge or specific heuristics. These features were designed to identify pertinent patterns and information in the data.

Local features: ORB [37] is an efficient feature detection and description algorithm designed to deliver a high computational speed, making it ideal for real-time computer vision applications. It serves as an alternative to SIFT and SURF, offering comparable performance with significantly reduced computational costs. ORB combines FAST for keypoint detection and BRIEF for descriptor generation, incorporating enhancements to ensure a robust performance. The process begins with keypoint detection using the FAST algorithm, which identifies corners by comparing the pixel intensities in a circular region. Scale invariance was achieved using a multiscale-image pyramid. The ORB then assigns an orientation to each keypoint using the intensity centroid method, which calculates the center of mass of the pixel intensities around the keypoint to ensure rotation invariance. Descriptors were computed using BRIEF, a binary encoding method that compares pixel intensities in pairs of pixels. The ORB enhances BRIEF by selecting the most discriminative binary tests and ranking keypoints using the Harris corner measure. These steps improve the robustness to noise and ensure computational efficiency. Binary descriptors are matched using the Hamming distance metric, which enables rapid and reliable matching, even in large datasets. The Hamming distance between two binary descriptors and each of length N × 32 bits, is computed as Equation (1):

(1) $$d_{H}\left(D_{1},\;D_{2}\right)=\;\sum_{i=1}^{N\times 32}\left|D_{1,i}-D_{2,i}\right|,\;\;\;$$

where

- $D_{1}$ and $D_{2}$ are the binary descriptors of two keypoints.
- $D_{1,i}$ and $D_{2,i}$ represent the i-th bit of descriptors $D_{1}$ and $D_{2}$, respectively.
- $\left|D_{1,i}-D_{2,i}\right|$ is the absolute difference between the bits at position i; it will be 1 if the bits are different (i.e., $D_{1,i}\neq D_{2,i}$) and 0 if the bits are the same (i.e., $D_{1,i}=D_{2,i}$).

The smaller the Hamming distance, the more similar the two descriptors are, and the more likely the key points are to match. This computationally efficient process is crucial for real-time applications, such as object recognition. The compact descriptor size of N × 32 ensures memory efficiency, making ORB particularly suitable for real-time applications such as object recognition. As shown in Table 3, samples of the normal and DFUs keypoints were extracted.

The BOF was then used to create a visual vocabulary by grouping the descriptors into a predefined number of clusters using algorithms such as k-means clustering. Each center represents a visual word cluster, and the set of these centers forms a visual codebook, which is a collection of visual words, each representing a cluster of similar feature descriptors. The size of the codebook determines the number of visual words and directly affects the granularity of the image representation. For a given image, the descriptors extracted in the first step are mapped to the closest visual words in the codebook based on a distance measure. This quantization step converts continuous feature descriptions into discrete labels corresponding to visual words.

**Figure 3:**
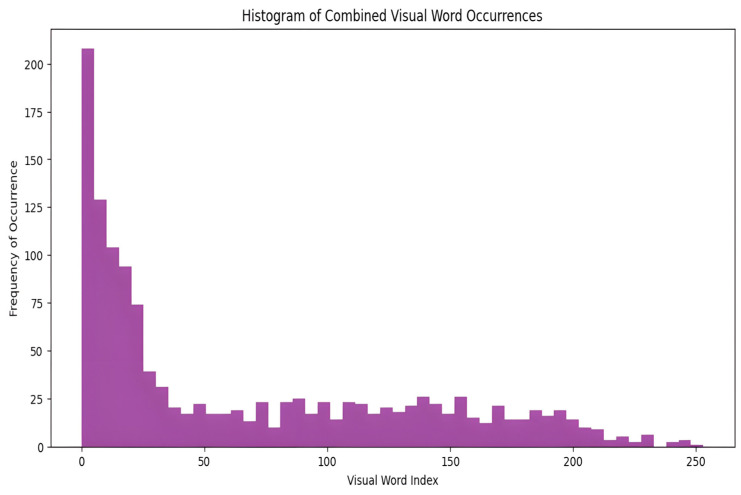
The distribution of visual words is identified in the images.

The image is then represented as a histogram of the frequencies of the visual words. The histogram captures the frequency of each visual word in the image and effectively summarizes its content. This histogram-based representation ignores the spatial layout, enhancing robustness against common transformations, such as rotation and scaling. Figure 3 illustrates a histogram showing the appearance of visual words in images from the dataset generated by applying the BOF technique to the ORB feature descriptors. This histogram serves as the foundation for training the classifier and for the actual classification of images. Consequently, each image was effectively encoded into a feature vector.

#### 3.2.2. Extracting DL-Based Features

We extracted DFU features from three pretrained DL models: ResNet-50, AlexNet [38], and EfficientNetB0 [39]. The aim of this study was to extract deep hierarchical features from thermal images of DFUs. We trained these models on more than one million images from the ImageNet database by preprocessing them in advance. By applying transfer learning, we adopted these models for our purposes without requiring extensive training on voluminous datasets. This significantly reduced the training time while improving the models’ effectiveness. DL models are designed to capture complex patterns and structures within images and provide advanced feature representations that improve the model’s ability to differentiate between DFUs and normal cases. The pretrained weights of VGG16 were specifically employed in the feature extraction method (frozen) to preserve generalization ability. The final proposed model tabulated the extracted features in an Excel file.

Finally, the proposed model leverages the advantages of both approaches by combining ORB feature extraction and DL-based feature extraction. This allows for a complete representation of thermal images for the diagnosis of DFUs in the future.

## 4. Proposed Method

Building upon the limitations identified in recent studies, we propose an efficient and robust hybrid method that integrates computational efficiency via ORB, robustness through BOF, and precision using DL.

**Figure 4:**
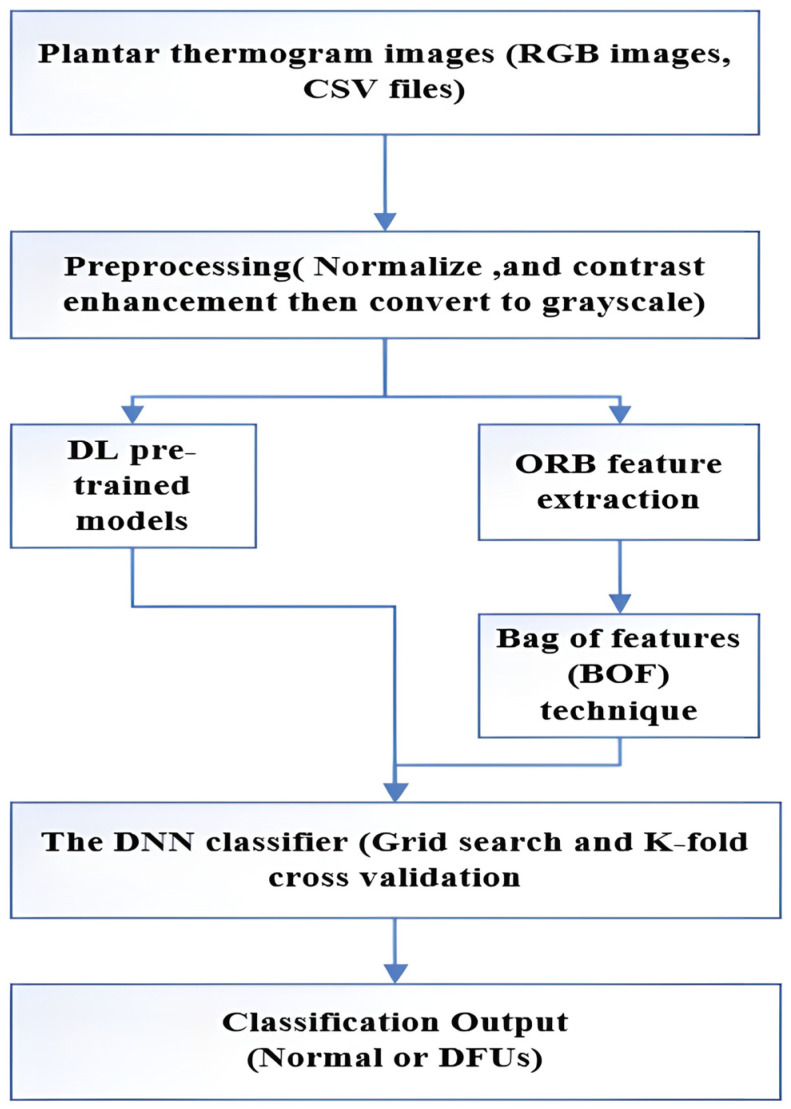
A flow diagram of the proposed model.

The proposed model builds on the foundational work of Alshayeji [21]. As shown in Figure 4, the proposed DNN model for diagnosing DFU from plantar thermogram images is laid out in a flow chart.

A foot thermal imaging database was selected to develop the proposed molecular analysis model, as thermal images can serve as accurate tools for assessing a patient’s condition before the development of DFUs. The distribution of body temperature provides different physiological data points in medicine, which aids in early diagnosis and prevention of disease progression. Our goal was to create a DNN model that could investigate the distribution of plantar temperatures in both groups and the extent to which these differences could be used to classify the feet of patients with DFU as normal.

We developed a novel diagnostic tool to assist medical professionals in taking preventive measures against foot ulcers. The database provides the corresponding data. csv files containing temperature data for each foot. Because the RGB images lacked temperature data, we used the corresponding CSV files that contained accurate thermal readings. The temperature values were converted into pixel intensities, resulting in images for further analyses. Thermal images from the control group exhibited distinctive temperature distribution patterns, whereas images from the diabetic group showed elevated temperatures in the central foot area, which gradually decreased toward the periphery. Thermal images of both feet were captured for each patient, along with their corresponding temperatures. csv files were analyzed individually. This approach allowed the model to identify unique temperature distribution patterns, as significant asymmetry was often observed between the two feet of the same patient.

Thermal camera images are impervious to external conditions, rendering them optimal for acquiring pristine, noise-free data. We employed normalization and contrast enhancement techniques on thermal images to prepare the training data for the modeling. Min–max normalization was used to scale pixel values between 0 and 255, ensuring standardized data and reducing training time. To further refine the contrast-enhancement process, we employed the CLAHE. CLAHE reduces excessive variance in uniform regions by applying a contrast reduction method, generating transformation functions for individual neighborhoods, and improving the overall image quality. The preprocessed images were then used in the subsequent feature extraction phase. In this stage, local features are extracted using the ORB algorithm, which identifies key points and generates descriptors for the images. Notably, this is the first study to apply the ORB and BOF techniques to thermal image analysis and DFU detection. Using the preprocessed thermal images, ORB successfully identified key points, and the corresponding descriptors were generated for further analysis, forming the foundation for the proposed DNN model trained on both handcrafted and DL features.

**Figure 5:**
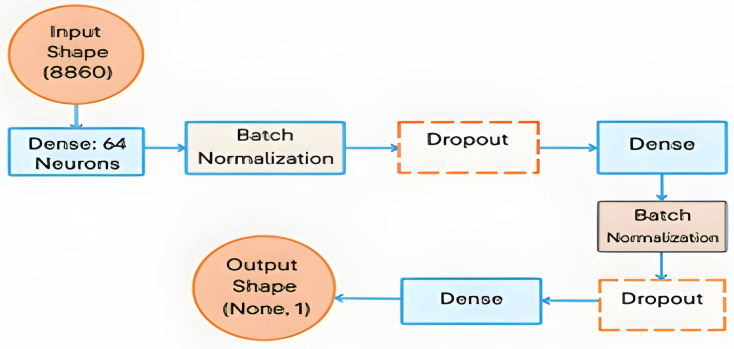
The proposed DNN model architecture.

The extracted ORB-based and DL features were combined to create a comprehensive feature set. Feature fusion explicitly consists of the concatenation of handcrafted ORB-based BOF histogram features with explicitly extracted deep learning features using pretrained CNNs (ResNet-50, AlexNet, and EfficientNetB0). This fusion of ORB-based and deep features ensures that the model benefits from both low-level textural details and high-level semantic information. Finally, these features were fed into the proposed DNN classifier. The proposed DNN model structure consists of two hidden layers, each containing 32 neurons, and uses a rectified linear unit (ReLU) as the activation function. Batch normalization layers were added after each dense layer to stabilize learning and improve convergence, and a dropout rate of approximately 47% was applied to prevent overfitting in the model. The output layer uses a sigmoid activation function, which is suitable for binary classification between DFU and normal cases, as shown in Figure 5. Overfitting was specifically addressed using other tools, such as batch normalization, dropout (33:43%), and 10-fold cross-validation, as mentioned in Section IV.

Several recent studies have explored the performance of vision transformers (ViTs) and CNN–transformer hybrid models in medical imaging tasks. Although these architectures have demonstrated strong classification capabilities, their practical applications remain constrained. Specifically, ViTs often require large, annotated datasets, high computational power, and extensive pretraining to match or surpass traditional CNN-based models. Matsoukas et al. [40] reported that ViTs underperform CNNs when trained from scratch and only show competitive performance when pretrained on large datasets using self-supervised methods. Similarly, Maurício et al. [41] emphasized the significant computational burden of ViTs, noting their limited suitability for real-time or low-resource clinical settings. In contrast, our proposed lightweight DNN model achieves high diagnostic accuracy while maintaining a fast inference time and minimal resource consumption, making it a more viable solution for early DFU screening.

To optimize the model performance, we used Optuna, an advanced framework for automated hyperparameter tuning. It dynamically explored optimal values for critical parameters, such as the number of neurons, dropout rate, batch size, and learning rate, streamlining the tuning process and eliminating the need for manual trial-and-error, as shown in Table 4.

The final DNN architecture consisted of two hidden layers with 64 neurons, ReLU activation functions, batch normalization, and a dropout rate of 33.43%, as determined through Optuna tuning.

The proposed optimized DNN model was trained using the DL-collected dataset and ORB-based features, combining both to generate a comprehensive feature representation. Ten-fold cross-validation was applied to evaluate the model’s robustness and mitigate overfitting. Each time, nine folds were used for training, while the remaining fold served as a validation set, ensuring that the model’s performance was evaluated on unseen data. When the DNN model was trained again using Optuna’s best hyperparameters on the whole dataset, it showed an elevated level of classification accuracy that could be used to diagnose DFU.

## 5. Results and Discussion

This section introduces the experimental platform and the performance metrics. Next, we conducted the main statistical analysis to validate the results. A comparative analysis with the existing literature was then performed to assess the contributions of this study, and the findings are presented. Finally, we discussed some limitations and observed statistical trends.

### 5.1. Experimental Platform and Performance Metrics

All experiments were conducted using an Intel Core i7 (11th Gen, 2.50 GHz, 8 cores) processor manufactured by Intel Corporation, Round Rock, TX, USA, and NVIDIA GeForce GTX 1660 SUPER GPU (1408 CUDA cores, 6 GB VRAM) manufactured by NVIDIA Corporation, Santa Clara, CA, USA. The software included Python 3.10, TensorFlow 2.x, OpenCV 4.7.0, and Optuna 3.3.0 for hyperparameter tuning.

The model performance was evaluated on the test data using different performance metrics: accuracy, precision, recall, F1-score, and ROC-AUC. The performance metrics were evaluated on the 20% test set using the standard definitions summarized in Table 5**.**

The optimal parameters identified using Optuna were applied during training (Table 4). Specific performance parameters were considered during the training stage to optimize the models used. These parameters included a batch size of 16, 64 neurons, a dropout rate of 0.3345952, an image count of 267, and an image size of 24 × 24. The DNN classifier underwent a training process spanning 28 epochs, with each epoch taking approximately 0.106 s on the GPU. Table 5 details the performance metrics utilized in this work, providing a clear view of the model’s capabilities and efficiency.

The variable TP represents the number of findings that the model classified as normal, whereas TN represents the number of samples that the model accurately identified as having DFU. FP denotes the number of samples that the model incorrectly identified as positive despite not having a DFU. FN denotes the number of samples that the model misclassified as DFUs, while they were, in fact, normal. The AUC-ROC is computed using the sum of indicator functions *I*(*P_i_* > *P_j_*) across all pairs of positive and negative samples, where *P_i_* is the probability assigned to a positive sample and *P_j_* to a negative sample. This metric reflects the model’s ability to distinguish between the two classes.

### 5.2. Statistical Results

#### 5.2.1. Preprocessing Results

**Figure 6:**
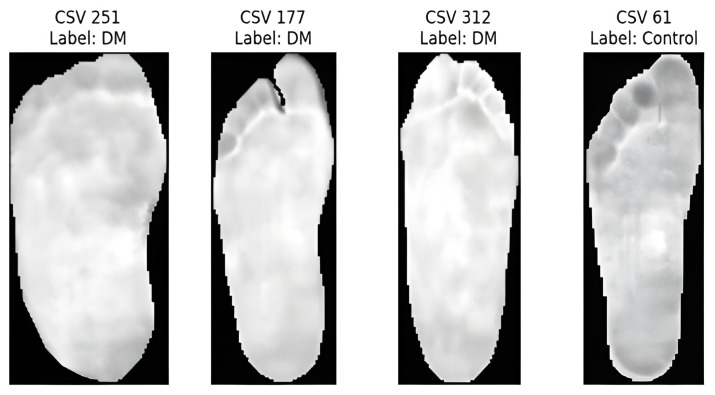
Samples of CSV temperature.

Two main image preprocessing methods were used for both the CSV temperature and RGB thermal images: min–max normalization and CLAHE. The temperature data in the CSV files were normalized on a scale of 0–255, resulting in grayscale images that depict temperature differences, as shown in Figure 6. We performed a grid search using several different CLAHE parameters, such as different cutoff boundaries and tile grid sizes, to determine the best configurations based on the Shannon entropy. This enhanced the contrast and information richness of the images, rendering them more suitable for subsequent analysis. Similarly, for RGB thermal images, grayscale transformation, and min–max normalization were performed before applying CLAHE. The grid search for CLAHE parameters produced optimal entropy values, demonstrating the greatest information gain as shown in Table 2.

#### 5.2.2. Feature Extraction Process

After preprocessing, we extracted ORB features from both the CSV and RGB images, filtered these features, and retained the images with valid feature descriptions. The combination of these feature sets was performed using the BOF method. ORB descriptors were clustered using k-means clustering to generate the histograms. These features were combined with DL features extracted from pretrained CNN models (ResNet50, AlexNet, and EfficientNetB0) using transfer learning. This combination results in a more comprehensive feature representation that captures both low- and high-level features.

#### 5.2.3. Classification Results

After implementing the BOF model, the extracted ORB and DL features were fed into the proposed DNN classifier. The dataset was split into training and testing subsets in an 80:20 ratio. The final evaluation was conducted on the test set using the best-performing configuration obtained via Optuna.

This study aimed to develop a model capable of accurately differentiating between thermal images of diabetic foot ulcers (DFUs) and normal feet. Local features, such as ORB, have proven to be efficient for keypoint detection in thermal images.

ORB features are robust to image noise, scale variation, and distortion, making them ideal for real-time medical diagnostic systems. The integration of handcrafted and deep features enables more accurate and robust classification.

**Figure 7:**
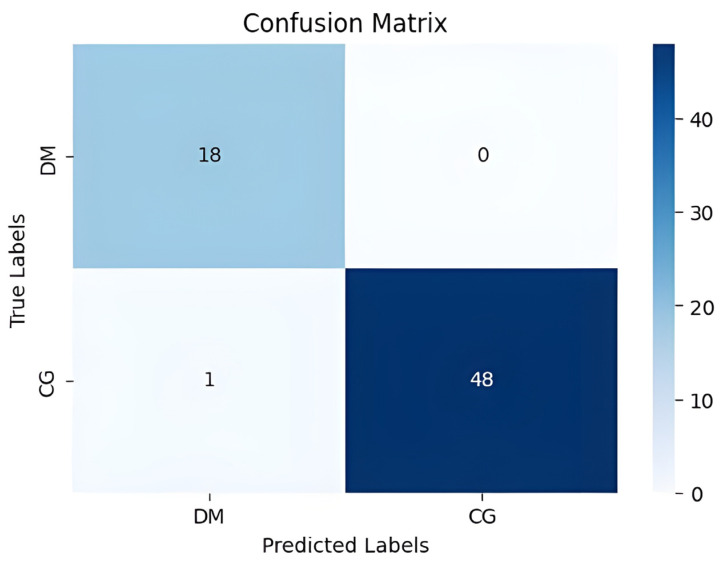
The confusion matrix visualizes classification performance.

**Figure 8:**
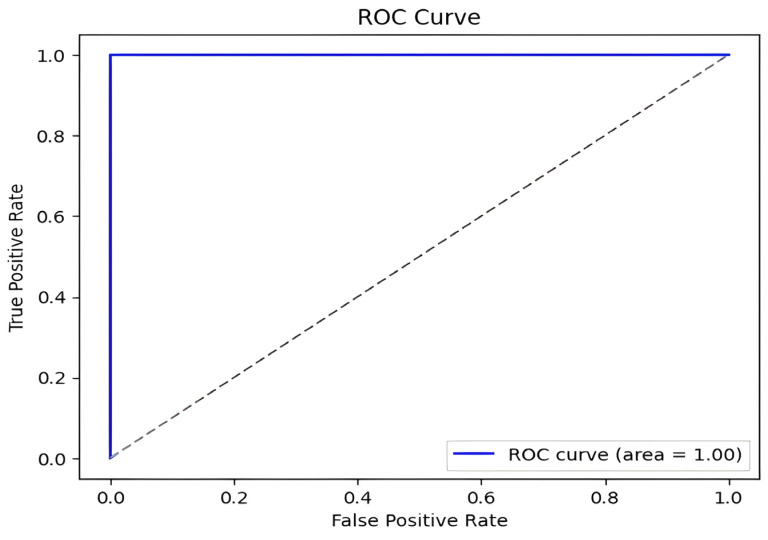
DFU classification model ROC curve.

Our method achieved the following results: 98.51% accuracy, 100% precision, 98.97% F1-score, and 100% AUC. As shown in Figure 7 and Figure 8, the confusion matrix performance showing correct classification rates for both normal and DFU cases and ROC curve are visualized (CG refers to normal and DM refers to DFUs). The detailed training and validation performance curves are provided in Appendix A.

#### 5.2.4. Ablation Study

An ablation study was conducted to validate the effectiveness of the hybrid feature extraction approach. We compared three models: ORB-only, DL-only, and Hybrid (ORB + DL). The results of this comparison are presented in Table 6.

The results underscore the diagnostic relevance of ORB-based features and demonstrate the enhanced accuracy achieved by combining handcrafted features with deep learning. Further performance metrics for each class are detailed in Table 7.

### 5.3. Comparative Analysis

The proposed model outperformed existing methods, achieving maximum accuracy (98.51%), precision (100%), and AUC (1.00). Compared with conventional methods [15,16,17,18,19,20,21], the proposed model offers superior real-time detection, increased accuracy, and resilient classification. The hybrid model combines ORB feature extraction with deep learning models, making it effective for DFU diagnosis. The results of this comparison are presented in Table 8.

Notably, the study referenced as [14] was intentionally excluded from Table 8. This decision was based on two considerations: first, the model reported in [14] achieved the lowest overall performance across all compared approaches (accuracy = 89.39%, AUC not reported); and second, the study predates 2020. Since this review aims to emphasize recent methodological advancements in DFU detection, only studies published from 2020 onward were included in the final benchmarking analysis.

In Table 8, we also explicitly compare our proposed model with existing state-of-the-art models in the literature [15,16,17,18,19,20,21]. Our approach outperformed these methods in terms of accuracy (98.51%), precision (100%), sensitivity (98.98%), and AUC (1.00). Compared to existing methods, the hybrid model of ORB-based handcrafted features and deep learning achieves a considerable increase in accuracy and robustness, which is attributed to enhanced feature extraction and computational efficiency.

**Figure 9:**
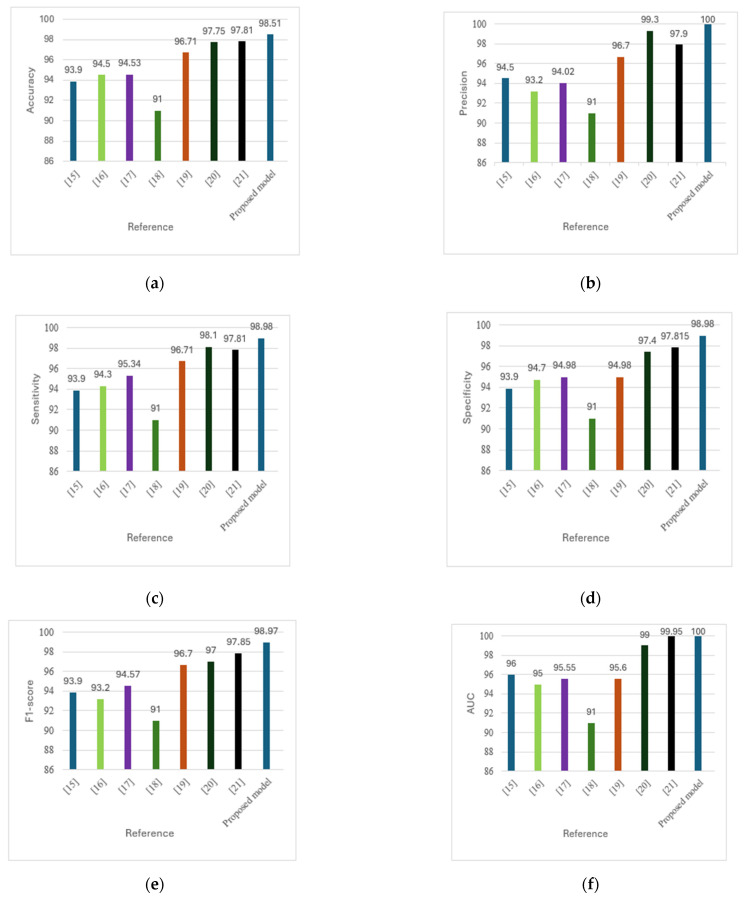
(**a**) Comparison of accuracy across methods. (**b**) Comparison of precision across methods. (**c**) Comparison of sensitivity across methods. (**d**) Comparison of specificity across methods. (**e**) Comparison of F1-score across methods. (**f**) Comparison of AUC across methods [15,16,17,18,19,20,21].

Figure 9 presents a comparative analysis of DFU detection methods across six performance metrics: (a) accuracy, (b) precision, (c) sensitivity, (d) specificity, (e) F1-score, and (f) AUC. The proposed model consistently outperformed existing methods [15,16,17,18,19,20,21] across all metrics, offering a superior balance of speed, accuracy, and computational efficiency. This enables real-time applicability and enhances clinical relevance by reducing misdiagnosis and improving the differentiation between affected and unaffected cases.

### 5.4. Limitations and Future Work

Despite the promising results, this study has several limitations. The model was trained and validated using a single-center dataset, which may affect its generalizability across different populations and imaging setups. Although techniques such as 10-fold cross-validation and stratified sampling were used, future studies should include external validation using data from multiple centers and devices to mitigate domain shift effects.

In addition, the current framework supports only binary classification (normal vs. DFU). We intend to expand the model to include multi-stage severity classification (grades 0–4), contingent on the availability of well-annotated datasets.

Future directions also include the development of a real-time user interface to facilitate clinical screening, enable early intervention, and reduce the burden of unnecessary referrals. Although the average inference time of 0.106 s per image demonstrates real-time feasibility, further optimization may be needed for deployment in lower-resource settings.

Ultimately, enhancing dataset diversity, incorporating multicenter collaborations, and adapting models to various hardware and patient demographics are essential next steps.

## 6. Conclusions

This study introduces an effective and lightweight diagnostic model for the early detection of diabetic foot ulcers (DFUs) by integrating handcrafted ORB features with deep representations from pretrained convolutional neural networks. To the best of our knowledge, this is the first study to apply ORB-based feature extraction in combination with deep learning for DFU detection using thermal imaging.

The proposed model achieved outstanding performance, including an accuracy of 98.51%, precision of 100%, sensitivity and specificity of 98.98%, F1-score of 98.97%, and AUC of 1.00. These results demonstrate its reliability and robustness in differentiating normal and DFU cases in the early stages.

With further development, this model can be deployed in a user-friendly clinical interface to support early diagnosis, reduce unnecessary hospital visits and facilitate prompt interventions. This is particularly valuable in resource-constrained or epidemic scenarios, where timely detection can significantly improve patient outcomes and reduce the burden on healthcare systems.

## Figures and Tables

**Table 1:** Summary of relevant related works (2018–2023).

Reference	Year	Method	Feature Extraction	Classifier	Accuracy	AUC	Limitations
[14]	2018	DWT + HOS-based	DWT, HOS	SVM	89.39%	N/A	Naturally, the study is limited in its dataset size, which may be too small for valid statistics and generalizability. It also uses only texture-based features and has no deep learning (DL) counterpart.
[15]	2020	DFUNet	CNN	CNN	93.9%	0.96	This study is constrained by having too few imbalanced cases and subjective manual annotations.
[16]	2020	DFU_QUTNet	CNN	CNN, SVM	94.5%	0.95	The model was developed using data from a single center only, and its diversity and external validity need to be validated in other institutions.
[17]	2020	Deep CNN for DFU classification	Automatic feature learning from thermograms	CNN	94.53%	0.9555	Did not incorporate handcrafted domain-specific features, reducing interpretability and potentially overlooking clinically relevant patterns.
[18]	2021	ML-based DFU detection	Thermogram texture/statistical features	SVM, Decision Trees	95.6%	0.9671	Relied solely on handcrafted features without leveraging deep learning, limiting scalability and robustness to complex variations in thermal patterns.
[19]	2022	TCI-based thermal asymmetry analysis	Thermal Change Index (TCI) descriptors	SVM, and KNN	91%	0.91	Focused only on asymmetry-based handcrafted features, without integrating deep learning representations, thus lacking hybrid strength and real-time deployment readiness.
[20]	2023	DFU_VIRNet	CNN	CNN	97.75%	0.99	The study lacks subject and imaging diversity details, affecting robustness. However, its fixed ROI size and computational burden may limit contextual exploration and application in low-resource circumstances.
[21]	2023	SIFT + SURF + Bag of Features (BOF)	SIFT, SURF, BOF	SVM	97.81%	99.95	The model’s high computational complexity may hinder real-time use.
Proposed	2025	Hybrid (ORB + DL)	ORB, BOF, ResNet50, EfficientNet	DNN	98.51%	1.00	Lightweight, robust to noise; real-time capability; novel feature integration.

N/A—not applicable.

**Table 2:** Illustrates a comparative analysis of preprocessing techniques on sample thermal images, highlighting the impact of normalization and contrast-enhanced image clarity and feature extraction efficiency.

	Normal	DFUs
Original Image	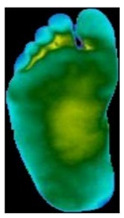	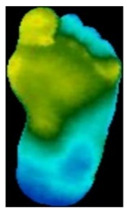
Normalized Image	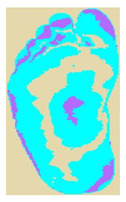	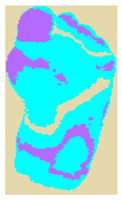
CLAHE	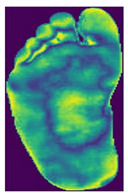	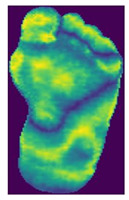

**Table 3:** Samples of normal and DFUs key points.

	Normal	DFUs
ORB key point visualization	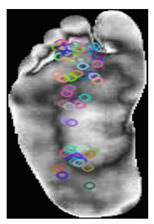	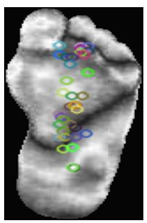
Total ORB key points	78	36

**Table 4:** The best-utilized hyperparameters to train the model.

The Best-Utilized Hyperparameters	
Neurons	64
Drop rate	0.334259522634211
Optimizer	Rmsprop
Batch size	16
Epochs	28

**Table 5:** Performance metrics [34].

Metric	Equation
Accuracy	(TP + TN)/(TP + TN + FP + FN)
Precision	TP/(TP + FP)
Sensitivity	TP/(TP + FN)
Specificity	TN/(TN + FP)
AUC-ROC	$\frac{\sum_{i=1}^{m}\sum_{j=1}^{n}I(P_{i}>P_{j})}{m\times n}$

**Table 6:** Comparison with trials on different sets of features.

Method	Accuracy	Precision	Sensitivity	AUC
ORB-only	88.0%	90.0%	85.0%	0.90
DL-only	95.5%	96.5%	95.0%	0.97
Hybrid (ORB + DL)	98.51%	100%	98.98%	1.00

**Table 7:** Best classification performance measures.

DNN Classifier	Class	Sensitivity	Specificity	Accuracy
	DFUs	0.9796	1.0000	0.9851
	Normal	1.0000	0.9796	0.985

**Table 8:** Clearly shows the enhanced performance of our hybrid approach of combining handcrafted ORB features with deep learning-based methods over the existing state-of-the-art techniques in terms of the accuracy, precision, sensitivity, and the AUC.

Ref.	Accuracy	Precision	Sensitivity	Specificity	F1-Score	AUC
[15]	93.9	94.5	93.9	93.9	93.9	96
[16]	94.5	93.2	94.3	94.7	93.2	95
[17]	94.53	94.02	95.34	94.98	94.57	95.55
[18]	91	91	91	91	91	91
[19]	96.71	96.7	96.71	94.98	96.7	95.6
[20]	97.75	99.3	98.1	97.4	97.0	99
[21]	97.81	97.9	97.81	97.815	97.85	99.95
Proposed	98.51	100.0	98.98	98.98	98.97	100.0

**Figure A1:**
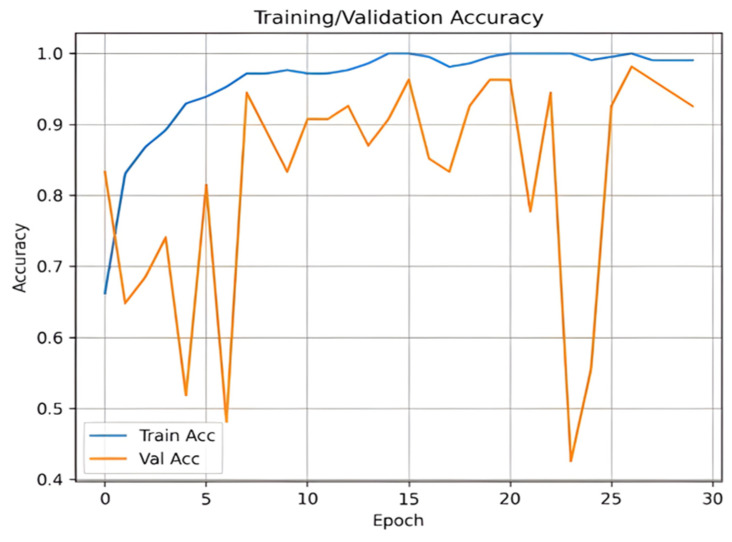
Training and validation accuracy curve.

**Figure A2:**
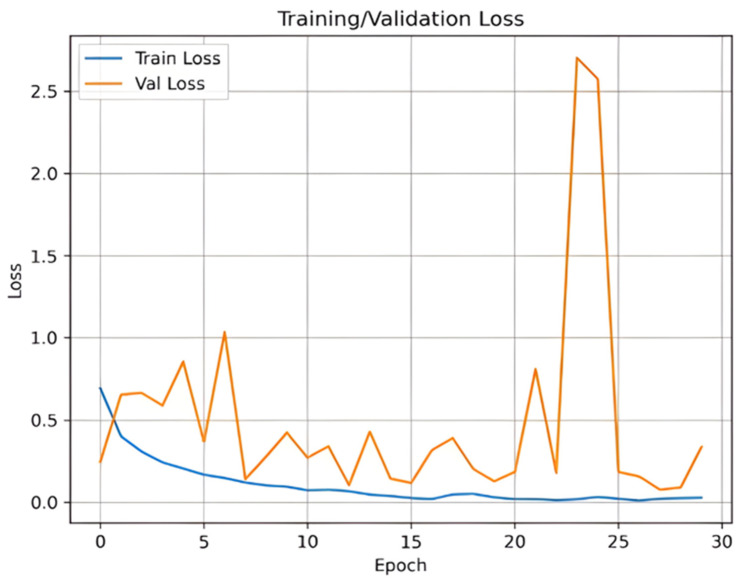
Training and validation loss curve.

## Data Availability

The data that support the findings of this study are openly available online in IEEE DataPort at https://ieee-dataport.org/open-access/plantar-thermogram-database-study-diabetic-foot-complications, (accessed on 1 December 2024).

## Appendix A. Training and Validation Performance

Figure A1 and Figure A2 illustrate the training and validation accuracy and loss curves, respectively. These curves demonstrate the model’s convergence behavior and generalization performance.

## Appendix B. Representative Test Predictions with Confidence Scores

This appendix includes representative thermal images from the test set, along with the model’s predicted class and associated confidence scores, as shown in Table A1. These examples highlight the model’s interpretability and reliability in classifying diabetic and normal cases.

**Table A1:** Representative DFU predictions with corresponding confidence scores and visualizations.

Thermal Image	Prediction Probability
	
	
	
	
